# Anatomic study suggests that the morphology of the plantaris tendon may be related to Achilles tendonitis

**DOI:** 10.1007/s00276-016-1682-1

**Published:** 2016-05-07

**Authors:** Łukasz Olewnik, Grzegorz Wysiadecki, Michał Polguj, Mirosław Topol

**Affiliations:** 1 Department of Normal and Clinical Anatomy, Interfaculty Chair of Anatomy and Histology, Medical University of Lodz, ul. Narutowicza 60, 90-136 Lodz, Poland; 2 Department of Angiology, Interfaculty Chair of Anatomy and Histology, Medical University of Lodz, Lodz, Poland

**Keywords:** Calcaneal tendon, Plantaris muscle, Mid-portion Achilles tendinopathy, Plantaris tendon

## Abstract

**Purpose:**

Achilles tendinopathy is a significant clinical lower limb issue observed in recent years. Neither the location nor the mechanism behind the pain has yet been sufficiently explained. Patients frequently experience pain on the medial side of the calcaneal tendon, and between 2 and 7 cm above the calcaneal tuberosity, which may suggests that the plantaris tendon plays an important role. The purpose of this study was to determine the anatomical relationships between the course of the plantaris tendon and the calcaneal tendon, as well as the type of insertion of the plantaris tendon.

**Methods:**

The tests were carried out on 50 randomized lower limbs (23 left and 27 right) fixed in 10 % formalin solution.

**Results:**

Five insertion types of the plantaris tendon were identified in relation to the calcaneal tendon: four with their insertion on the calcaneal tuberosity (Types 1, 2, 3, 5), while the fifth (Type 4) had its insertion in the crural fascia. In addition, two variants of the course of the plantaris tendon were identified, the most common being termed Variant A, in which the tendon crosses the space between the gastrocnemius and the soleus muscles, thus reaching the medial crural region, and is located on the medial side of the calcaneal tendon (84 % cases). The course of the Variant B is similar to the course of the Variant A, but upon leaving the space located between the gastrocnemius and soleus muscle, it turned to the medial crural region and ran directly anterior to the calcaneal tendon (12 %). The plantaris muscle was found to be absent in two lower limbs (4 %). The most frequent insertion type of the plantaris tendon into the calcaneal tuberosity is fan-shaped, occurring on the medial side of the Achilles tendon (Type 1–44 % cases).

**Conclusion:**

The course of the plantaris tendon and its mobility range in relation to the calcaneal tendon may be likely to affect the occurrence of pains in the lower medial part of the leg (Achilles tendinopathy).

## Introduction

The plantaris muscle (PM) is characterized by a short, slim muscle belly and a long tendon [12]. It originates on the popliteal surface of the femur, above the lateral condyle and on the capsule of the knee joint. The length of the muscle belly ranges between 5 and 10 cm [9]. It is situated between the popliteal muscle, located anterior to it, and the lateral head of the gastrocnemius muscle, located posterior to it [4]. The belly becomes a long, thin tendon, as it runs towards the medial crural region, passing between the medial head of the gastrocnemius muscle and the soleus muscle [15]. Upon leaving this section, it most commonly rests along the medial edge of the calcaneal (Achilles) tendon, ending on the calcaneal tuberosity. The distal course of the plantaris tendon and its insertion are characterized with high anatomic variability [1, 13, 20]. In some rare cases, the PM has been observed to be absent [2].

The PM is a biarticular muscle, which is particularly active at the plantar flexion of the foot, which occurs with simultaneous full straightening of the knee joint. Most of the injuries of this muscle occur in combination with a break in the continuity of the gastrocnemius, the soleus muscle or the anterior cruciate ligament and very often calcaneal tendon [9, 18]. In recent years, there was an increase re-rupture of Achilles tendon, which may be associated with increasing physical activity in modern societies [10, 16]. Therefore, the optimal treatment of both acute Achilles tendon ruptures and Achilles tendon re-ruptures also remains of great clinical importance [10, 18].

Some authors suggest that the course of the plantaris tendon may have an impact on Achilles tendinopathy as well as etiopatogenesis of pain in the medial crural region [3, 7, 11, 17, 19, 20].

The purpose of this study was to determine the anatomic relationships between the course of the plantaris tendon and the calcaneal tendon, and to identify the types of insertion of the plantaris tendon.

## Materials and methods

Fifty randomized and isolated (23 left and 27 right) lower limbs were obtained from adult cadavers (24 female and 26 male) and fixed in a 10 % formalin solution before examination. The cadavers were the property of the Normal and Clinical Anatomy Department, Medical University of Lodz, Poland. Lower limbs with visible, macroscopically detectable, anomalies were excluded from the study. The study procedure was approved by the Medical University of Lodz Bioethical Commission (agreement no. RNN/182/15/KE).

A classical anatomical preparation comprising the crural and foot area was carried out. Upon dissection, the morphology of the plantaris tendon was assessed, together with the location and area of its insertion to the calcaneal tuberosity.

The next stage included the anthropometric measurements of the tendon. The distance between the calcaneal tuberosity and the point of fan-shaped insertion of the tendon was measured. In addition, the width and thickness of the tendon were measured at the extension point (Tables 1, 2, 3). An electronic digital caliper was used for all measurements (Mitutoyo Corporation, Kawasaki-shi, Kanagawa, Japan). Each measurement was carried out twice with an accuracy of up to 0.1 mm.

**Table 1:** Plantaris tendon width at the extension point

Type	The maximum PT width at the extension point (mm)	The minimum PT width at the extension point (mm)	The mean PT width at the extension point (mm)	Standard deviation (mm)
1	4.1	1.3	2.85	1.1
2	2.0	1.1	1.7	0.3
3	2.8	2.1	2.4	0.3
4	3.65	2.8	3.2	0.6
5	6.1	2.65	4.3	1.3

*PT* plantaris tendon

**Table 2:** Plantaris tendon thickness at the extension point

Type	The maximum PT thickness at the extension point (mm)	The minimum PT thickness at the extension point (mm)	The mean PT thickness at the extension point (mm)	Standard deviation (mm)
1	1.2	0.9	1.0	0.1
2	1.6	0.4	1.0	0.3
3	1.7	1.2	1.4	0.2
4	1.0	0.9	1.0	0.1
5	1.3	0.6	0.9	0.2

*PT* plantaris tendon

**Table 3:** Distance between the calcaneal tuberosity and the plantaris tendon extension point

Type	The maximum distance between the CT and the PT extension point (mm)	The minimum PT distance between the CT and the PT extension point (mm)	The mean distance between the CT and the PT extension point (mm)	Standard deviation (mm)
1	65.2	34.3	49.4	12.9
2	61.5	39.2	46.9	7.8
3	53.4	41.4	49.1	5.4
4	73.1	61.5	67.3	8.2
5	6.1	2.65	4.3	1.3

*PT* plantaris tendon*, CT* calcaneal tuberosity

Basic descriptive statistics were calculated for the collected dimensions.

## Results

Five types of insertion were found for the plantaris tendon into the calcaneal tuberosity based on its shape, its relation to the calcaneal tendon and the exact point of insertion in the calcaneal tuberosity.

**Fig. 1:**
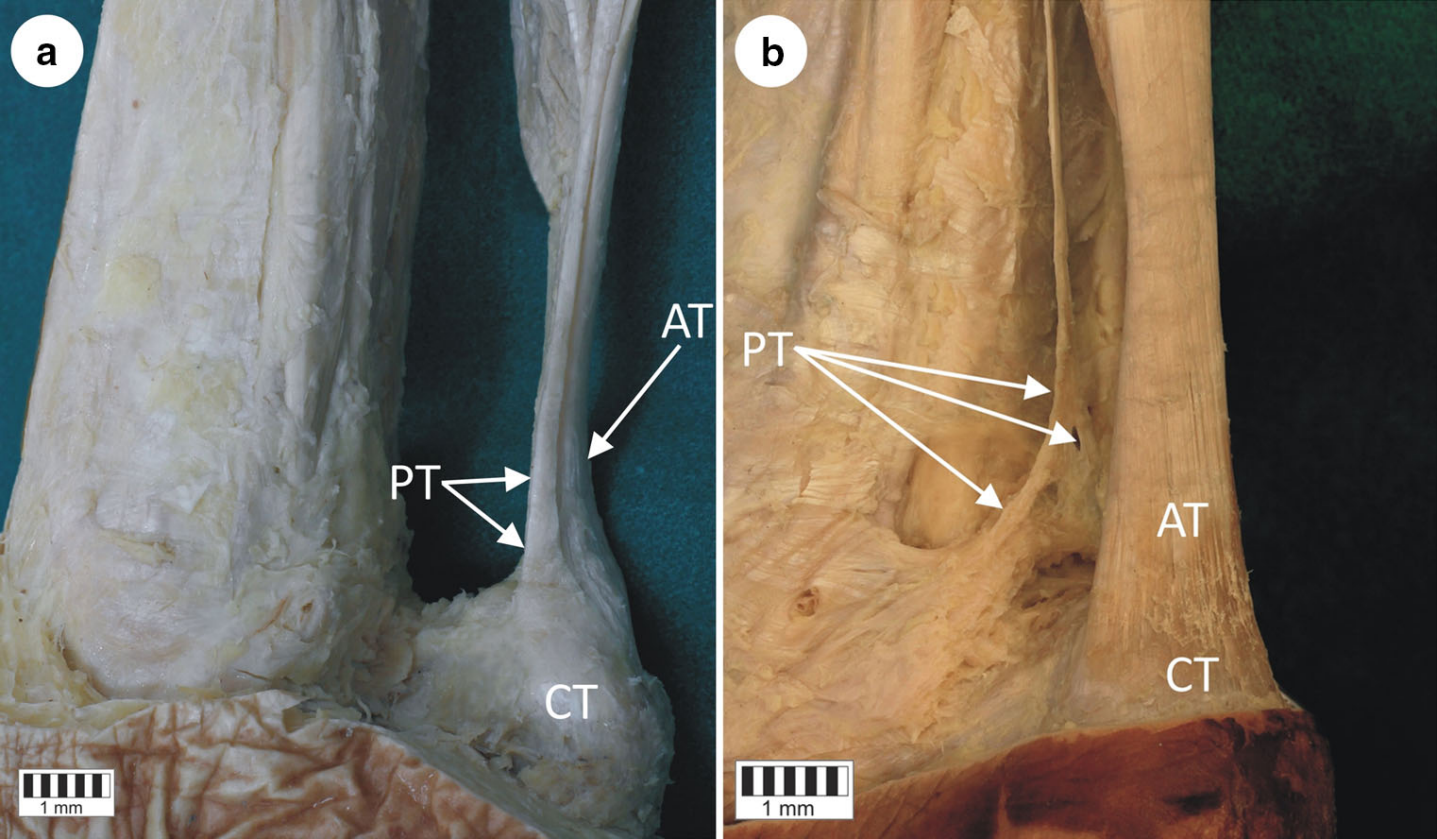
Tendon of the plantaris muscle. **a** Type 1 of plantaris tendon insertion to the calcaneal tuberosity (the most common). Medial view of the right leg. The course of the plantaris muscle tendon visible on the medial part of the calcaneal tendon. **b** Type 4 of the plantaris tendon insertion (the less common). Posteromedial view of the right leg. The figure shows the atypical insertion of the plantaris tendon to the deep fascia of the leg. The lack of the insertion located on the calcaneal bone is visible. *AT* Achilles tendon, *CT* calcaneal tuberosity, *PT* plantaris tendon

**Fig. 2:**
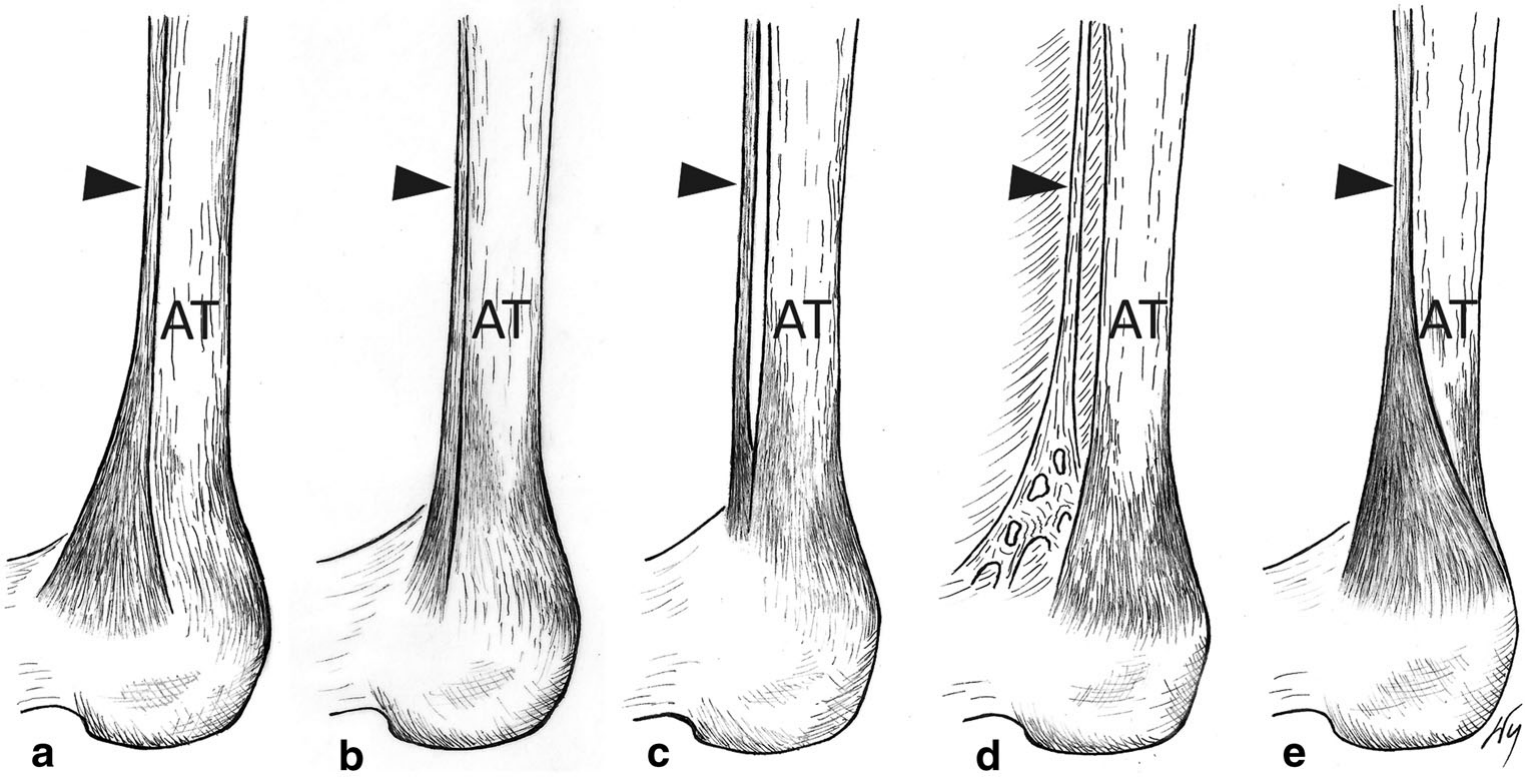
Tendon of the plantaris muscle. Schematic representation of the major types of the plantaris tendon insertion. Medial view of the right leg. **a** Type 1 (44 %), **b** Type 2 (18 %), **c** Type 3 (8 %), **d** Type 4 (4 %), **e** Type 5 (22 %). *Black arrowhead * plantaris tendon

Type 1 is characterized by a wide, fan-shaped insertion to the calcaneal tuberosity on the medial side of the Achilles tendon (Figs. 1a, 2a). It was observed in 22 lower limbs (44 %): 11 right and 11 left. The mean width of the tendon was 2.85 mm (1.3–4.0 mm) and thickness 1.02 mm (0.9–1.2 mm) at the extension point, while the mean distance between the calcaneal tuberosity and the plantaris tendon insertion was 49.4 mm (34.3–65.2 mm) (Tables 1, 2, 3).

Type 2 is characterized by insertion to the calcaneal tuberosity, along with the calcaneal tendon; however, a slight branching of the plantaris tendon occurs, which does not take the characteristic fan shape observed in Type 1 (Fig. 2b). It was detected in 9 lower limbs (18 %): 5 right and 4 left. This type was characterized by a mean tendon width of 1.7 mm (1.1–2.0 mm) at the extension point, a mean thickness at this point of 1 mm (0.4–1.6 mm), and a mean distance between the calcaneal tuberosity and the plantaris tendon extension point of 46.9 mm (39.2–61.5 mm) (Tables 1, 2, 3).

Type 3 is characterized by insertion at the calcaneal bone, anterior to the calcaneal tendon (from 0.8 to 2.1 mm) (Fig. 2c). This was present in 4 lower limbs (8 %): 3 right limbs and 1 left. It was characterized with a mean tendon width of 2.4 mm (2.1–2.8 mm) at the extension point, a mean thickness at this point of 1.4 mm (1.2–1.7 mm), and a mean distance between the calcaneal tuberosity and the plantaris tendon extension point of 49.1 mm (41.4–53.4 mm) (Tables 1, 2, 3).

Type 4 is characterized by the insertion not being located on the calcaneal tuberosity but rather in the deep crural fascia. It has no direct ‘communication’ with the Achilles tendon but runs 2.3–2.4 mm anterior to it (Figs. 1b, 2d). Such a type was detected in two lower limbs (4 %): one right and one left. This type was found to have a mean tendon width of 3.2 mm (2.8–3.65 mm) and a mean thickness of 1 mm (0.9–1.0 mm) at the extension point, and a mean distance between the calcaneal tuberosity and the plantaris tendon extension point of 67.3 mm (61.5–73.1 mm) (Tables 1, 2, 3).

Type 5 is characterized with a very wide insertion encircling the posterior and medial surfaces of the Achilles tendon (Fig. 2e). This type of insertion occurred in 11 limbs (22 %): 7 right and 4 left. This type was found to have a mean tendon width of 4.3 mm (2.65–6.1 mm) and a mean thickness of 0.9 mm (0.6–1.3 mm) at the extension point, while the mean distance between the calcaneal tuberosity and the plantaris tendon extension point was 4.29 mm (2.65–6.1) (Tables 1, 2, 3).

**Fig. 3:**
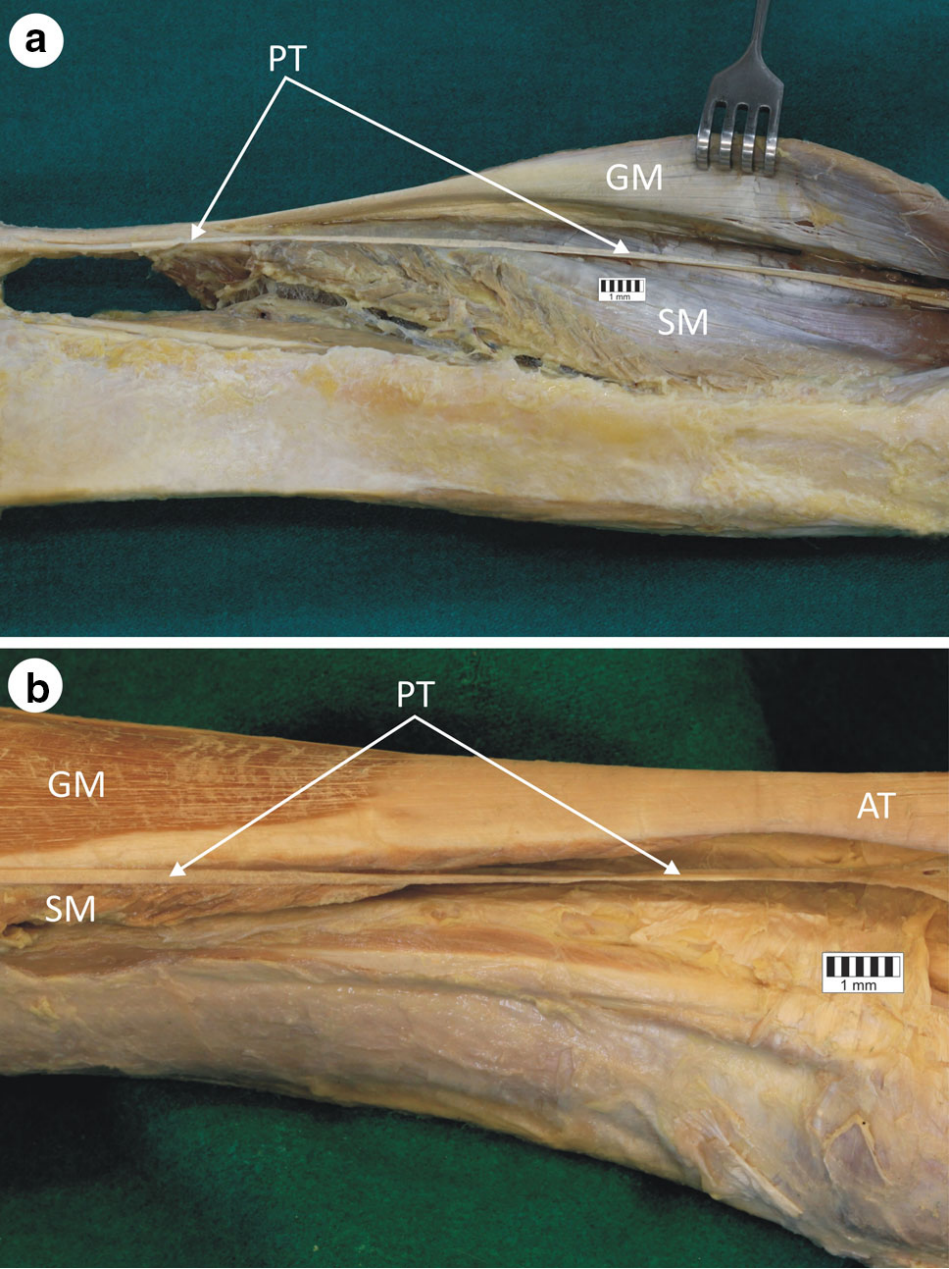
Variable anatomical relationships of the plantaris tendon. **a** Variant A of the plantaris tendon course. **b** Variant B of the plantaris tendon course. *AT * Achilles tendon, *GM* gastrocnemius muscle, *SM* soleus muscle, *PT* plantaris tendon

An analysis of the course of the plantaris tendon from its beginning at the belly of the muscle in relation to the calcaneal tendon revealed the presence of two course variants. Variant A was noted most frequently, occurring in 42 lower limbs, or 84 % of cases (Fig. 3a). In this variant, the plantaris tendon was located on the medial side of the Achilles tendon, and its course initially crossed the space between the gastrocnemius and the soleus muscle, to eventually reach the medial crural region. This variant included examples of Types 1, 2, and 5 of PM insertion to the calcaneal tuberosity (Table 4), with the tendon closely adhering to the Achilles tendon in Type 2, but running separately in Type 1.

**Table 4:** Dependence between the insertion type and the plantaris tendon course variant

PT course variant	Type of insertion of PT	PT course characteristics
A	1	PT crosses the space between GM and SM, and runs on the medial part of the leg, medial to Achilles tendon PT runs separately from Achilles tendon
A	2	PT crosses the space between GM and the SM, and runs on the medial part of the leg, medial to Achilles tendon PT closely adheres to Achilles tendon, within single paratendon
B	3	PT crosses the space between GM and SM, and descends along the medial part of the leg, and next runs anterior to Achilles tendon PT has insertion anterior to the calcaneal tendon
B	4	PT crosses the space between the GM and SM, and runs on the medial part of the leg, and next runs anterior to Achilles tendon PT inserts to deep crural fascia of the leg
A	5	PT crosses the space between GM and SM, and runs on the medial part of the leg, medial to Achilles tendon PT has a very wide insertion over the Achilles tendon

*PT* plantaris tendon, *GM* gastrocnemius muscle, *SM* soleus muscle

Variant B of the course occurred in the cases where insertion Types 3 and 4 were formed by distal insertion of the plantaris tendon (Table 4). The initial course was at first identical to that of Variant A, however, upon leaving the space located between the gastrocnemius and the soleus muscle, it turned to the medial crural region and directly ran anterior to the Achilles tendon. This type of plantaris tendon course occurred in six lower limbs, which constituted only 12 % of cases (Fig. 3b).

The PM was found to be absent in two limbs, one right and one left (4 %).

## Discussion

In the examined material, five modes of insertion of the plantaris tendon to the calcaneal tuberosity were identified. In addition, the location of the plantaris tendon in relation to the Achilles tendon also varied. Our findings partly resemble those of Cummins et al. [5], who differentiated four types of plantaris tendon insertion in the calcaneal tuberosity while dissecting 200 lower limbs in the crural and foot region.

Cummins et al. [5] characterise Type I of the plantaris tendon insertion as a fan-shaped insertion into the medial aspect of the superior calcaneal tuberosity for the insertion of the calcaneal tendon. This type was found to be the most common, occurring in 47 % of tests. Similarly, the frequency of Type I in the present study was found to be 44 %.

Type II of the plantaris tendon insertion, according to Cummins et al. [5], is characterized by inserts to the calcaneus, occurring 0.5–2.5 cm anterior to the medial border of the calcaneal tendon. Cummins et al. [5] note that this type may additionally insert into the flexor retinaculum of the foot and to the fascia covering the medial part of the calcaneal bone. They also report it as the second most commonly occurring type, being present in 36.5 % of samples. In contrast, this type was only found in 8 % of cases in the present study, and the Cummins et al. [5] classification does not include the option of insertion to the flexor retinaculum of the foot or to the fascia.

Type III, according to Cummins et al. [5], is a broad insertion investing the posterior and medial surfaces on the adjacent terminal calcaneal tendon. This type was present in 12.5 % of their samples, compared to 22 % within ours.

Type IV, according to Cummins et al. [5], is an insertion into the medial border of the calcaneal tendon at a level 1–16 cm proximal to the point at which the calcaneal tendon is inserted into the calcaneus. Cummins et al. [5] believe that this type of plantaris tendon may directly insert into the calcaneal bone. They found it to be the rarest type of insertion, being present in only 4 % of cases. Similarly, our present findings include no such examples of this type of insertion.

In addition to the four types described by Cummins et al., our findings distinguish two further types, defined in our results as Type 2 and Type 4: Type 2 is characterized by a common insertion to the calcaneal tuberosity of the plantaris and the calcaneal tendons, which closely adhere to each other, while Type 4 is characterized by a lack of ‘communication’ with the Achilles tendon and the fact that it does not insert into the calcaneal tuberosity, but rather to the deep crural fascia. Type 4 was found to occur in two lower limbs in our tests (4 % of cases).

Based on a study of 107 lower limbs, Sterkenburg et al. [20] distinguish as many as nine different points of plantaris tendon insertion: (1) medial onto calcaneus; (2) medial, fan-shaped onto calcaneus; (3) medial onto calcaneal tendon; (4) medial with thin slips onto calcaneus; (5) anteromedial onto calcaneus; (6) anteromedial, fan-shaped onto calcaneus; (7) posteromedial, fan-shaped onto calcaneus; (8) anterior onto calcaneus; (9) deep fascia.

Five of the insertion types defined by Sterkenburg et al. resemble the types identified in the present study. Namely, the anteromedial onto calcaneus type is, to a certain extent, like our Type 2, while the anteromedial, fan-shaped corresponds to our Type 1. We have found further similarities between the posteromedial, fan-shaped onto calcaneus type, which corresponds to our Type 5. The anterior onto calcaneus type is similar to our Type 3. The deep fascia was the rarest type identified by Sterkenburg et al. test, and it resembles our Type 4. The other four types distinguished by Sterkenburg et al. were not found in our dissection material.

Sterkenburg et al. [20] found that the plantaris tendon ran separately from the Achilles tendon in 96 of 107 studied cases, but were closely connected to each other in the other 11. Similarly, in the present study, while the plantaris tendon and calcaneal tendon were separate in 41 of 50 cases, they were joined in the remaining 9 lower limbs.

Sterkenburg et al. [19] observe that the close connection between the plantaris tendon and the calcaneal tendon was located at the level of the mid-portion of the Achilles tendon, the level at which the pains are detected by patients with Achilles tendinopathy. Due to the fact that a substantial portion of patients suffering from the Achilles tendinopathy experience pain located between 2 and 7 cm above the calcaneal tuberosity on the medial crural side [6], presumably the patients with Variant A of the PM tendon course and Type 2 of the insertion of the tendon may experience pain on the medial crural side. To analyze the above thesis more thoroughly, however, some ultrasound imaging should be carried out on patients with diagnosed Achilles tendinopathy.

The PM was found to be absent in two of the lower limbs examined in the present study (one right and one left), representing 4 % of cases. Similarly, Harvey et al. [8] report the presence of a PM in 532 of 658 studied lower limbs: it being absent in the remaining 19 %. The PM was absent in 6.67 % of cases in a dissection of 750 lower limbs by Daseler and Anson [2], and between 7 and 20 % of cases described by Simpson et al. [14]. However, both Aragão et al. [2] and Sterkenburg et al. [20] report the PM to be present in all examined lower limbs: 107 in the case of the latter.

It is worth emphasizing that the present study describes anatomical variants of the plantaris tendon course in relation to the calcaneal tendon that have not been described previously. These are marked as Variant A and Variant B of the plantaris tendon course (Table 4).

## Conclusions

Five types of insertion of the plantaris tendon were distinguished in the studied material, four with their insertion point at the calcaneal tuberosity (Type 1, 2, 3, 5), and one (Type 4) with its insertion in the deep crural fascia. Of the two identified course variants (A and B), the most common was Variant A (84 % cases), where the tendon, located on the medial side of the calcaneal tendon, initially crossed the space between the gastrocnemius and the soleus muscle before running onto the medial crural region. The most common variant of PM insertion in the calcaneal tuberosity was found to be the fan-shaped insertion on the medial side of the Achilles tendon (defined as Type 1), which occurred in 44 % cases.
